# Transgenerational epigenetic inheritance of axonal regeneration after spinal cord injury

**DOI:** 10.1093/eep/dvad002

**Published:** 2023-01-17

**Authors:** Andy Madrid, Reid S Alisch, Elias Rizk, Ligia A Papale, Kirk J Hogan, Bermans J Iskandar

**Affiliations:** Department of Neurological Surgery, University of Wisconsin—Madison, Madison, WI 53719, USA; Department of Neurological Surgery, University of Wisconsin—Madison, Madison, WI 53719, USA; Department of Neurosurgery, Penn State Children’s Hospital, Hershey, PA 17033, USA; Department of Neurological Surgery, University of Wisconsin—Madison, Madison, WI 53719, USA; Department of Anesthesiology, University of Wisconsin—Madison, Madison, WI 53719, USA; Wisconsin Alzheimer’s Institute, University of Wisconsin—Madison, Madison, WI 53719, USA; Department of Neurological Surgery, University of Wisconsin—Madison, Madison, WI 53719, USA

**Keywords:** DNA methylation, epigenetics, CNS regeneration, folic acid, folate, one carbon metabolism

## Abstract

Human epidemiological studies reveal that dietary and environmental alterations influence the health of the offspring and that the effect is not limited to the F1 or F2 generations. Non-Mendelian transgenerational inheritance of traits in response to environmental stimuli has been confirmed in non-mammalian organisms including plants and worms and are shown to be epigenetically mediated. However, transgenerational inheritance beyond the F2 generation remains controversial in mammals. Our lab previously discovered that the treatment of rodents (rats and mice) with folic acid significantly enhances the regeneration of injured axons following spinal cord injury *in vivo* and *in vitro*, and the effect is mediated by DNA methylation. The potential heritability of DNA methylation prompted us to investigate the following question: Is the enhanced axonal regeneration phenotype inherited transgenerationally without exposure to folic acid supplementation in the intervening generations? In the present review, we condense our findings showing that a beneficial trait (i.e., enhanced axonal regeneration after spinal cord injury) and accompanying molecular alterations (i.e., DNA methylation), triggered by an environmental exposure (i.e., folic acid supplementation) to F0 animals only, are inherited transgenerationally and beyond the F3 generation.

## Introduction

**Figure 1:**
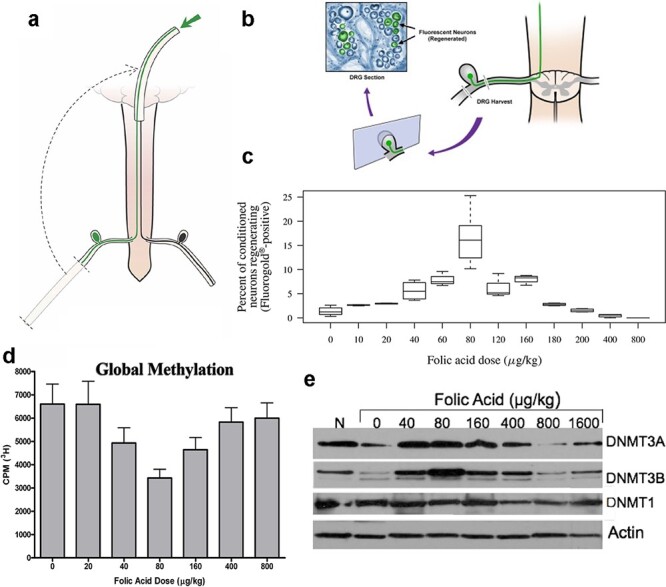
Biphasic responses following increasing folic acid supplementation. (a) A spinal cord injury paradigm used to study the effects of folic acid supplementation on spinal axon regeneration *in vivo.* Animals were treated with intraperitoneal (i.p.) doses of folic acid, starting 3 days prior to the injury and given daily for 2 weeks. A sciatic nerve graft is implanted at the site of bilateral C3 dorsal column transection (arrow) to provide a permissive environment for the transected spinal axons to grow. At 2 weeks, a fluorescent tracer is placed at the free end of the graft (arrow). (b) At 48 hours, the lumbar DRG, in which the cell bodies of the transected axons reside, are harvested, sectioned, and inspected with fluorescence microscopy. The percent of fluorescent neurons is calculated. In this model, spinal axons on the side of the sciatic nerve harvest (conditioned side) are more likely to grow axons into the graft. (c) Percent axon regeneration follows a biphasic curve with increasing doses of folic acid, peaking at 80 µg/kg and returning to baseline levels by 800 µg/kg. (d, e) Global levels of DNA methylation measured in spinal cord tissue by a radioactive assay (note: the lower radioactive counts correspond to higher methylation levels) (d), and the expression of the *de novo* DNMTs DNMT3a and 3b, but not the maintenance DNMT1, measured in the same tissue by Western analysis (e), follows identical biphasic curves in response to folic acid supplementation (CPM: counts per minute; N: no spinal cord injury control; reproduced from previous publications [1, 3])

**Figure 2:**
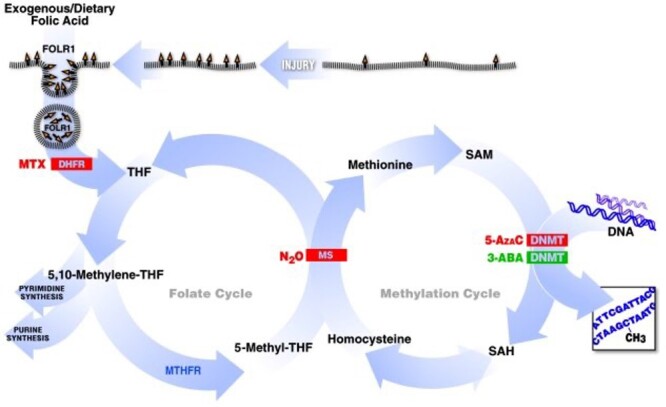
The folate and methylation pathway. Folic acid enters the cell through the FOLR1, which is upregulated with injury. The folic acid molecule is then converted into the active tetrahydrofolate (THF) form by DHFR. This allows the eventual production of nucleotides and certain amino acids, as well as the transfer of the methyl group into the methionine-methylation cycle. The latter occurs through the B12-dependent MS step. Subsequently, S-adenosylmethionine (SAM) is the substrate used by the methyltransferase enzymes for the methylation reactions. The inhibition of FOLR1, DHFR, MS, and DNMT suppresses CNS regeneration. In turn, the activation of DNMT enhances CNS regeneration (MTX: methotrexate; 3-ABA: 3-aminobenzamide; SAH: S-adenosylhomocysteine) (reproduced from previous publication) [3]

Nearly 20 years ago, our neuroscience laboratory discovered that parenteral folic acid administered for 3 days before spinal cord injury enhances the regeneration of sensory spinal axons in a peripheral nerve placed at the site of a sharp spinal cord injury (Fig. 1a, b) [1]. Surprisingly, the folic acid dose dependency was biphasic with a small effect at low doses, a peak effect at 80 µg/kg, and a loss of enhanced axon regeneration at higher doses with no observed toxicities (Fig. 1c). When presented with these unforeseen data, we noted that biphasic dose dependencies have precedence elsewhere in pharmacology and are often authentic rather than artifactual. Our anesthesia laboratory had just discovered that nitrous oxide, a specific and potent inhibitor of the vitamin B12 cofactor in methionine synthase (MS), combined with two inactivating polymorphisms in the gene encoding 5,10-methylene tetrahydrofolate reductase (MTHFR), was responsible for multiple central nervous system (CNS) lesions and the death of a small child arising from the impaired availability of methyl groups required for multiple synthetic pathways [2]. This first evidence of a two-hit pharmacogenomic syndrome, in which proteins encoded by a mutant gene and the drug target are different in a shared pathway, highlighted the potentially deleterious consequences of interference with the evolutionary ancient folate–cobalamin cycles responsible for the generation of methyl substituents in all cells of the body (Fig. 2). With a shared focus on the pleotropic and profound effects that bracket single-carbon pathway interventions via a long-standing collaboration between the two laboratories, we sought to identify the precise steps of the folate cycle that mediate the post-injury CNS regeneration phenotype. Folate agonists and antagonists that alter the targeted enzymatic reactions disclosed a relationship between axon regeneration and the methionine-methylation arm of the folate pathway, with consequent changes in deoxyribonucleic acid (DNA) methylation (Fig. 1d, e) [3]. In 2003, Waterland and Jirtle showed that dietary methyl supplementation of agouti mouse a/a dams with single-carbon donors including folic acid alters the phenotype of their Avy/a offspring via increased CpG methylation at the Avy locus and that methyl supplementation to F0 parents may have deleterious influences on epigenetic gene regulation in F1 progeny [4] (CpGs are stretches of DNA with the sequence cytosine and guanine nucleotides with the “p” representing the linking phosphate. CpGs are often found in repeated sequences called CpG islands. DNA Methylation occurs at the C5 position of the cytosine to form 5-methylcytosine, frequently in CpG islands). Shortly thereafter, Skinner and his laboratory reported altered DNA methylation patterns in the germ line, in correlation with the transmission of reduced sperm number and viability and increased male infertility to the F4 generation after the exposure of just the F0 generation to endocrine disruptors [5]. Motivated by these intriguing observations, we asked: Does F0 administration of folic acid promote the regeneration of sensory spinal axons after spinal cord injury in F3 progeny and beyond, with no exposure to folic acid supplementation in the interval generations? In the present narrative review, we condense the findings of hundreds of controlled experiments in thousands of animals over 15 years that persuade us that this is the case.

### Folate and Axonal Regeneration after Spinal Cord Injury in a Single Generation

#### *Folic Acid Prior to Spinal Cord Injury Enhances Axonal Regeneration* In Vivo

Whereas the developing mammalian CNS is able to generate and extend neurons [6], the differentiated CNS is among the most refractory tissues to heal after injury [7]. Folate levels are elevated in the embryonic CNS and diminish significantly at birth [8]. Because embryonic pathways may be experimentally reactivated in the injured adult CNS to promote axonal regeneration [9], we hypothesized that the administration of folic acid to support single-carbon donor pathway function improves the capacity of adult CNS axons to regenerate after injury. In the spinal cord regeneration model, the cervical dorsal columns comprising sensory neurons are sharply transected, and a 2-cm autologous sciatic nerve graft harvested from the left hind limb of the animal is implanted at the injury site [10]. Axons in the dorsal columns and the sciatic nerve share a cell body in the dorsal root ganglia (DRG). Injury of the peripheral axon of the DRG by harvesting the sciatic nerve graft enhances the growth of both the peripheral sciatic nerve axons and the DRG’s central dorsal column axons. Thus, the peripheral nerve injury triggers the DRG to regenerate centrally directed spinal cord axons. We found that pre-injury treatment with intraperitoneal (IP) folic acid at 80 µg/kg markedly increases the proportions of dorsal column sensory neurons that extend axons into the peripheral nerve graft [1, 3, 11]. The combination of peripheral nerve injury and IP folic acid supplementation is additive with a ∼10-fold increase in the number of regenerating axons on the side ipsilateral to the peripheral nerve harvest site [1, 3]. These data supported the use of the combined unilateral sciatic nerve and bilateral dorsal column injury model for the subsequent regeneration, molecular, and biochemical experiments. When folic acid doses were escalated from 20 to 800 µg/kg, spinal sensory axon regeneration increased with the dose amount until it reached a maximum at 80 µg/kg, with diminished regeneration seen thereafter with each incremental dose, and a return to a baseline level of minimal regeneration at 800 µg/kg, in keeping with a biphasic, inverted U curve dose dependency (Fig 1c) [1, 3].

#### *Folic Acid Enhances Axonal Regeneration via Neuronal Rather Than Glial Mechanisms* In Vitro

To test the contribution of glial vs. neuronal factors to folic acid–mediated axonal regeneration after injury, the left lumbar DRG of rats with injury to the left sciatic nerve and both dorsal columns were harvested after folic acid pretreatment, dissociated, and placed in a culture medium permissive of axonal growth to establish a pure neuronal culture devoid of glia (Fig. 1b) We observed a significant increase in axonal regeneration of isolated neurons in culture from the folate-treated animals compared to vehicle controls over a 48-hour period. These results indicate that folate-induced post-injury axonal regeneration is independent of glial–neuronal interactions [3].

#### *Folic Acid–Induced CNS Axon Regeneration* In Vivo *Requires a Functional Folate Pathway*

Transport of folates across epithelial membranes and into cells is mediated by three transporters [12]: the folate receptor (FOLR1), the proton-coupled folate transporter (SLC46A1), and the reduced folate carrier (RFC, SLC19A1). While RFC is ubiquitously expressed with low affinity for folic acid and other folates, FOLR1 expression is confined to specific epithelial tissues and is a high-affinity receptor. After the entry via FOLR1, folic acid is activated by dihydrofolate reductase (DHFR) before undergoing a series of enzymatic conversions to produce nucleotides and amino acids and to support the transfer of methyl groups into the methionine-methylation cycle via MS to produce S-adenosylmethionine. S-adenosylmethionine is a substrate for methyltransferase enzymes, including the DNA methyltransferases (DNMTs), in methylation reactions (Fig. 2). The folate pathway is essential for nucleotide synthesis and methylation of DNA, ribonucleic acid (RNA), and proteins. To examine which steps of the folate pathway underlie the heightened axonal regeneration phenotype, we first showed that spinal cord injury increases the expression of FOLR1, but not RFC, in spinal cord tissues and that a heterozygous knockdown of *Folr1* (*Folr1*^+/−^) reduces the spinal axonal regeneration capacity compared to wild-type controls in response to folic acid supplementation [3]. Proton-coupled folate transporter has only recently been recognized as a folate transporter and was not tested in these experiments [12]. Next, we specifically inhibited DHFR, MS, and the DNMTs using methotrexate, nitrous oxide, and 5-azadeoxycytidine, respectively, all of which blocked spinal neurons from regenerating axons after injury. Conversely, the activation of DNMT with 3-aminobenzamide in the absence of folic acid supplementation also increased CNS axonal regeneration [3]. These data indicate that the axon regeneration phenotype depends on a functional folate receptor and biochemical pathway.

#### *The Folate Pathway Mediates Spinal Axon Regeneration via DNA Methylation* In Vivo

While DNMT1 maintains patterns of DNA methylation, DNMT3A and DNMT3B participate in *de novo* DNA methylation. To test whether the DNMTs modulate folic acid–mediated axonal regeneration, we used immunoblot assays to measure the protein expression with escalating folic acid doses. We observed no difference in DNMT1 levels following spinal axon injury and folic acid supplementation. However, the protein levels of DNM3A and DNMT3B were significantly suppressed by injury and restored to baseline with folic acid supplementation. Moreover, with increasing doses of folic acid, DNMT3A/B expression followed a biphasic curve in parallel with that observed in spinal axon regeneration (Fig. 1d, e) [3]. These results indicate that folic acid supplementation results in robust modulations in the expression of genes and in the DNA methylation pathway that contribute to the axonal regeneration phenotype.

Altered expression of DNMT3A/B observed in our animal model denotes that DNA methylation is involved in augmented spinal axon regeneration after injury. To test this hypothesis, we used a radioactive assay to measure methyl group incorporation and observed that spinal cord injury generates a 40% decline in global DNA methylation levels in spinal cord tissues, a decrement that is prevented by folic acid supplementation. In turn, axon regeneration with escalating doses of folic acid tracks a biphasic dependency in DNA methylation that parallels the biphasic curves observed with spinal axon regeneration and Dnmt3a/b protein expression (Fig. 1e). These results indicate that the magnitude of DNA methylation sustains folic acid–mediated axonal regeneration.

### Ancestral Folate and Transgenerational Inheritance of Enhanced Axon Regeneration after Spinal Cord Injury

#### *Ancestral Folate Promotes* In Vivo *Axonal Regeneration in Serial Generations of Untreated Progeny*

Human epidemiologic data show that parental exposures to environmental stimuli alter DNA methylation in several generations of unexposed progeny in parallel with major changes in health indices [13]. Because the folate pathway is the sole methylation source in the body [14], and in view of the data by Skinner et al. [5], we theorized that DNA methylation–linked improvements in CNS axon regeneration may not be limited to the treated individuals but may also be transmitted to the subsequent untreated progeny. To test this hypothesis, mating pairs of outbred Sprague-Dawley rats were treated with IP folic acid at the optimal dose of 80 µg/kg. The treated and mated pairs generate lineages of male and female offspring to be phenotyped for enhanced spinal axon regeneration. Amazingly, the supplementation of F0 ancestors with folic acid engenders improved spinal axonal regeneration in the untreated F1 and F2 progeny. Because the exposure of a gestating (F0) dam to a drug or other experimental condition also directly exposes the F1 embryo and its F2 gamete via an “intergenerational transmission”, “transgenerational” inheritance of a trait requires the transmission of the trait to at least the F3 generation (or F2 generation if only F0 males are treated) [15, 16]. Consequently, untreated F2 progeny were mated to procreate F3 and F4 generations with the enhanced regeneration phenotype in support of transgenerational inheritance of this trait [17].

With these data in hand, we conducted a multiplicity of control experiments to test the validity of the transgenerational data. First, mating F0 breeding pairs were treated with parenteral 5-methyltetrahydrofolate (L-methylfolate) folate congener to control for specificity of methyl donor agonist. This folate agonist does not require DHFR conversion to be activated. Also, a non-parenteral diet supplemented with folic acid, betaine, choline, and vitamin B12 (Harlan Laboratories Diet 7017-MS) was also provided to mating F0 breeding pairs. Both the parenteral L-methylfolate and the oral methyl-supplemented diet independently enhanced spinal axon regeneration to at least four successive untreated generations (F1–F4). These results indicate that the transgenerational axonal regeneration phenotype is specific to methyl donor agonism but not to its specific analog or route of administration.

**Figure 3:**
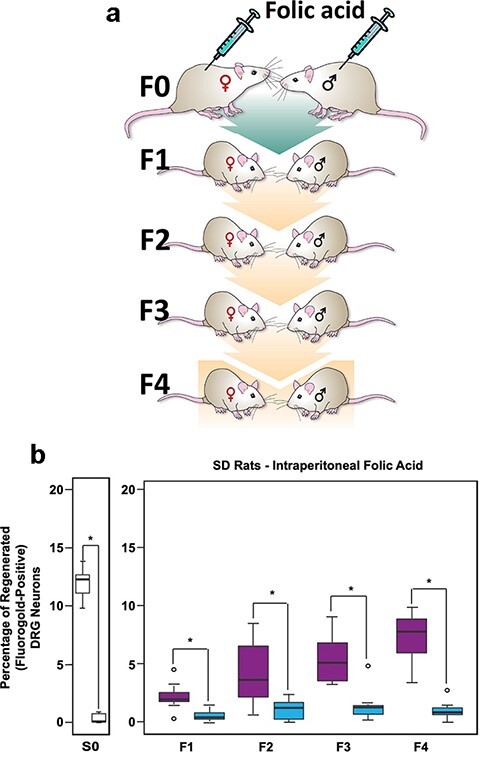
Ancestral folic acid enhances the regeneration of injured CNS axons in multiple generations of untreated progeny. (a) A mating pair of animals (F0) was treated with intraperitoneal folic acid (80 µg/kg) or vehicle distilled de-ionized water (DDI) starting 2 weeks before breeding and continuing in females until weaning and in males until pregnancy was assured. Four generations of progeny were bred without treatment. (b) Box plots show that the folic acid supplementation of progenitors enhances percent spinal axon regeneration in untreated F1–F4 male progeny (left boxes) compared to controls (right boxes). Single-generation control animals (S0) with direct exposure to folate supplementation exhibit ∼12% regeneration. Identical results were obtained using various routes of administration, breeding schemes, and two distinct genera (not shown) (reproduced from previous publication) [17]

Next, inbred Fischer rats were used to test whether the transgenerational post-injury–enhanced axonal regeneration phenotype is specific to a rat strain (Fig. 3a). We observed identical results in the untreated F1–F4 Fischer rat progeny (Fig. 3b) [17]. Furthermore, folate-induced enhanced regeneration of spinal axons in untreated F3 descendants was evident in both *in vivo* and *in vitro* experiments [17]. Most tellingly, we discovered that the enhanced transgenerational regeneration effect of folate administration is present in two distinct mammalian genera, i.e. rat and mouse [17]. To our knowledge, this is the first evidence of transgenerational epigenetic inheritance (TEI) of an identical trait, either adverse or beneficial, that is shared across a genus boundary after an identical exposure. Statistical modeling of these data indicates that the transgenerational inheritance of enhanced spinal cord regeneration after spinal cord injury cannot be due to random selection of a founder effect or by a Mendelian genetic model [17], further confirming a role of the methylome in the inheritance of this adaptive phenotype.

Together, these data indicate that the TEI of the enhanced axonal regeneration phenotype is not an artifact of folate analog, route of administration, *in vivo* vs. *in vitro* experimental models, outbred or inbred rodent strain, or rodent genus.

#### The Axonal Regeneration Phenotype Is Co-inherited with Differentially Methylated Regions of Genomic DNA and Altered RNA Transcription

**Figure 4:**
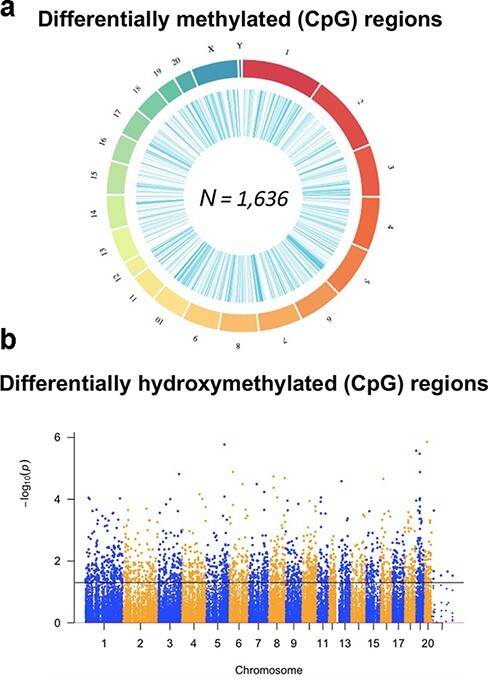
Ancestral folic acid results in transgenerational perturbations in DNA methylation and hydroxymethylation. (a) A circus plot depicts the representative locations of genome-wide DMRs identified when comparing spinal cord tissue derived from folic acid lineage F3 male offspring to F3 male vehicle control offspring. The outer circle represents the various chromosomes in the rat genome. The inner circle represents the relative location of the DMRs. (b) A Manhattan plot depicts the relative location of genome-wide regions tested for 5hmC abundance for the various chromosomes (x-axis) in the rat genome. The y-axis depicts the -log10 value of the *P*-value when comparing spinal cord tissue derived from folic acid lineage F3 male offspring to F3 male vehicle control offspring. Dots above the dashed line represent differentially hydroxymethylated regions (reproduced from previous publication) [17]

To test molecular mechanisms by which the regenerative phenotype is inherited, we administered inhibitors of DNA methylation (5-aza-2ʹ-deoxycytidine) and histone deacetylase (trichostatin A) to the F3 generation and beyond. Both blocked the pro-regenerative effect of ancestral folate [3]. Alterations in DNA methylation measured via methylated DNA immunoprecipitation and in the transcriptome measured via messenger RNA (mRNA) sequencing (RNA-seq) were compared in the F3 progeny of folic acid–treated and control F0 lineages. We identified 908 differentially methylated regions (DMRs; ≥5 CpGs, >10% differential, *P*-value < 1e-5) and 782 differences in mRNA transcript levels in spinal cord tissues between lineages with and without ancestral F0 folic acid. Transgenerational changes in both DNA methylation and mRNA expression levels were observed in 13 genes [17]. Methylated DNA immunoprecipitation does not distinguish between 5-methylcytosine (5mC) and 5-hydroxymethylcytosine (5hmC). To distinguish the contributions of 5mC and 5hmC to axonal regeneration, genomic DNA from the identical tissues was subjected to whole-genome DNA methylation sequencing (Fig. 4a). Axonal regeneration–related modifications in DNA methylation levels comprising 5mC and 5hmC were ascertained at 2260 DMRs [18]. Using selective enrichment, hydroxymethylated DNA was sequenced to identify regions of the genome with 5hmC. A total of 658 differentially hydroxymethylated regions included differentially hydroxymethylated regions in genes with recognized links to axonal regeneration (e.g. *Ank3, Cntf*) (Fig. 4b). A comparison of the DNA methylation and hydroxymethylation data to mRNA dataset sequences in samples from the same F3 spinal cord tissues exhibited 65 genes with differential methylation/hydroxymethylation and gene expression, in correlation with transgenerational inheritance of the enhanced axonal regeneration phenotype. These data indicate that ancestral F0 treatment with folic acid results in lasting, transgenerational conversions in the DNA methylome that are co-inherited with the post-injury axonal regeneration phenotype.

## Perspectives

Single-carbon pathways support the capacity of injured spinal cord axons to regenerate after injury. In turn, the beneficial effects of folate administration require a fully functional methylation cycle. The effects of folate on regeneration of spinal afferent neurons are biphasic and dose-dependent in correlation over a wide dose range with global and gene-specific DNA methylation and with the activity of *de novo* DNMTs. The folate-enhanced axon regeneration phenotype is surprisingly heritable via non-Mendelian patterns of inheritance in tandem with differential regions of DNA methylation, hydroxymethylation, and differential RNA expression.

Folate (vitamin B_9_) is an essential nutrient required for a diversity of enzymatic reactions and processes in a broad spectrum of biological pathways, including DNA replication, nucleotide and amino acid synthesis, metabolism, and methylation reactions [14]. As the sole source of methylation in eukaryotes, the folate pathway is essential and necessary in providing functional methyl groups for DNA, RNA, histones, and non-histone proteins, suggesting that epigenetic mechanisms that govern CNS axon regeneration may be shared by other biological systems, including those with regenerative or healing functions, such as liver, skin, and bone. While our investigations have been limited to the adult CNS, one of the most refractory tissues to regenerate axons and restore function after injury, it is likely that the epigenetic effects of folate span other tissues and body functions.

The molecular mechanisms underlying non-genomic inheritance are incompletely resolved, and the concept of mammalian TEI remains open to controversy [19]. Accordingly, our ongoing and future work addresses experimental TEI via *in vitro* fertilization, examines the potential role of other epigenetic mechanisms including histone modification and small non-coding RNAs, and accounts for DMRs that escape erasure after fertilization and embryonic development. As well, we are testing the contributions of sex of transmission, DNA hydroxymethylation, pharmacokinetics (i.e. concentrations of folate congeners in different physical compartments over time intervals after administration), different genera, and other injury models that may comprise benefits to progeny after ancestral single-carbon supplementation.

Although we have only just begun to glimpse the mechanisms that underlie the beneficial effects of folates on axon regeneration after injury, we note that neuronal trauma appears to dysregulate folate pathway receptors and enzymes while simultaneously increasing the requirement for enzyme cofactors and methylation of gene promoters. A subset of genes with increased promoter methylation participate in conventional neuronal function pathways, whereas others such as *Creb1, Dscam, Emb*, and *Runx3* [18] participate in embryonic development, possibly reflecting the reversion of injured neurons to a “growth-competent” state [22]. Surprisingly, our data show that the effects of neuronal injury and folate supplementation are not limited to the injured F0 animal. To the contrary, heritable adaptive mechanisms are induced that foreshadow adaptive responses to an environmental insult that may endow descendant generations for a shared exposure. Twenty years ago, it would not have been possible to predict where our mutual interests in neuroscience, pharmacology, and genomics would lead, and how profoundly our understanding of inheritance between cells in mitosis, meiosis, and evolution has changed in view of experiments designed to control for TEI confounders in the interval. Our views of phylogeny, ontogeny, and the genesis of each and every trait have undergone radical reconfiguration [15,20,21, 23–25]. Most of all, we are captivated by the tempos and magnitudes of the methylome’s plasticity and the epigenome at large.

## Data Availability

No new data is provided in this article. All data and figures were previously generated and published elsewhere.
